# Tele-assessment of Mobility and Balance is Reliable and Safe for Individuals with Chronic Stroke – A Guideline for a Systematic Physical Evaluation

**DOI:** 10.63144/ijt.2025.6710

**Published:** 2025-12-12

**Authors:** Bruna Nascimento Zanfir da Silva, Camila Pinto, Caroline Santos Figueiredo, Thainara Cruz da Rosa, Katherine Lee Hsieh, Aline Souza Pagnussat

**Affiliations:** 1 Graduate Program in Rehabilitation Sciences, Universidade Federal de Ciências da Saúde de Porto Alegre (UFCSPA), Brazil; 2 Movement Analysis and Rehabilitation Laboratory, Universidade Federal de Ciências da Saúde de Porto Alegre (UFCSPA), Brazil; 3 Graduate Program in Health Sciences, Universidade Federal de Ciências da Saúde de Porto Alegre (UFCSPA), Brazil; 4 Department of Physical Therapy, Georgia State University (GSU), Atlanta, Georgia, USA; 5 Department of Physical Therapy, Universidade Federal do Rio Grande do Sul (UFRGS), Brazil

**Keywords:** Neurological rehabilitation, Stroke, Telehealth, Telemonitoring, Telerehabilitation

## Abstract

Despite the growing use of telehealth in stroke rehabilitation, it remains unclear whether online assessments (i.e., tele-assessment) can match the accuracy of in-person evaluations. Given the high prevalence of stroke and its significant impact on mobility, precise assessment is essential. This study evaluated the reliability and safety of a tele-assessment protocol for mobility and balance in individuals with chronic stroke, using the OMPEPE guideline. Twenty-five participants underwent in-person and synchronous tele-assessment (Timed Up and Go, Five Times Sit-to-Stand, and Functional Reach tests) within 24 to 48 hours. One month later, the same physiotherapist and a second investigator independently scored asynchronous video recordings. Results showed strong agreement between online and in-person assessments, with excellent intra- and inter-rater reliability (ICC > 0.90). Most participants had moderate to severe motor impairment. These findings suggest that tele-assessment is a reliable and safe approach when systematically planned, providing an effective solution for monitoring individuals with chronic stroke conditions when in-person healthcare is not feasible.

In recent years, there has been a significant increase in the use of telehealth services driven by rapid technological advancements and the growing need for more accessible healthcare delivery models (Zheng et al., 2022). Telehealth encompasses various services provided through telecommunications technologies, including therapy, education, monitoring, and teleconsultation (Darkins, 2000). Within this broad field, tele-assessment has emerged as a particularly promising strategy for remotely evaluating patients’ clinical and functional status (Kidholm et al., 2024). It involves the transfer of audiovisual data between the clinician and the patient and can be conducted either synchronously (in real-time) or asynchronously (through recorded data) (Chan et al., 2018).

Parallel to this growth, the clinical adoption of telerehabilitation has expanded considerably, enabling clinicians to remotely assess motor function in individuals with neurological conditions using validated and reliable tools (O’Neil et al., 2023). Among these conditions, stroke stands out due to its global prevalence and its profound impact on mobility, independence and quality of life (Feigin et al., 2024). Early and continuous rehabilitation is crucial to improve function and prevent long-term disability (Li et al., 2024). However, even after completing structured neurorehabilitation programs, many individuals continue to experience persistent functional limitations, underscoring the need for ongoing monitoring and regular follow-up assessments throughout the recovery process (Gil-Salcedo et al., 2022).

Recent evidence has further supported the feasibility and validity of remote motor assessments in this population. For instance, a recent study demonstrated excellent inter- and intra-rater reliability when applying the Berg Balance Scale via both synchronous and asynchronous tele-assessment in individuals with stroke (Önal et al., 2025). Similarly, studies involving healthy participants have also shown good intra-rater reliability for mobility tele-assessment (Bird et al., 2022). Despite these encouraging results, there remains a notable gap in literature regarding other key outcomes, such as functional mobility, functional lower limb strength, and dynamic balance in individuals with stroke. Moreover, the absence of standardized assessment protocols continues to raise concerns about the consistency, reproducibility and accuracy of remote evaluations.

Therefore, this study aimed to develop and examine the reliability and safety of a standardized tele-assessment protocol for evaluating mobility and balance in people with chronic stroke impairments, using the Timed Up and Go Test (TUG), Five Times Sit-to-Stand Test (5TSTS), and Functional Reach Test (FRT). These tests were selected based on their extensive clinical use and feasibility for remote administration.

## Methods

This observational study was conducted in accordance with the Guidelines for Reporting Reliability and Agreement Studies (GRRAS) (Kottner et al., 2011). Ethical approval was obtained from the Institutional Human Research Ethics Committee of the Federal University of Health Sciences of Porto Alegre (Approval no.: 45137321.5.0000.5345).

### Participants

The sample size was calculated using the WinPepi software (http://www.brixtonhealth.com/pepi4windows.html), based on data from a previous study (Russell et al., 2002). A total of 25 participants was estimated to be sufficient to achieve a minimum agreement of r = 0.90, with 90% power and a 5% significance level.

Participants were recruited for convenience sampling between December 2020 and October 2022 through advertisements posted on social media and in hospitals located in Porto Alegre, Brazil, and the surrounding metropolitan area. Individuals were considered eligible if they met the following inclusion criteria: age over 18 years; clinical diagnosis of chronic stroke (>6 months) with mild, moderate, or severe hemiparesis, according to the Fugl-Meyer Assessment (FMA) (Daly et al., 2011); no cognitive impairment (minimum score of 20 points in the Mini-Mental State Examination) (Folstein et al., 1975); ability to walk independently for at least 3 meters with or without walking devices; having a cell phone, tablet, or computer with a camera and internet access where evaluations could be carried out; basic knowledge of videoconferencing (participant or another person assisting them); a full-time caregiver or family member available during the online assessments; and signed the informed consent form. Individuals who had a clinical diagnosis of secondary musculoskeletal disorders affecting performance, any unstable or severe illness interfering with assessments, peripheral neurological or musculoskeletal conditions impacting balance or gait, a history of neurosurgery or orthopedic surgery resulting in gait limitations, or significant uncorrected visual deficits were excluded from the study.

### Procedures

**Figure 1:**
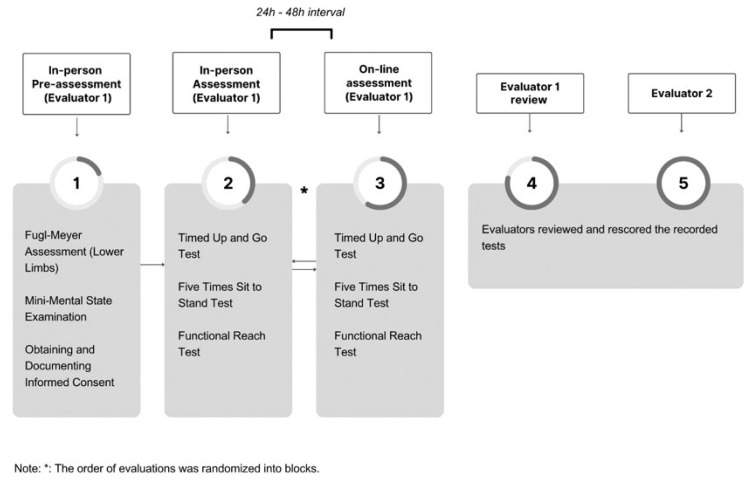
Assessment Flow Chart

Participants were assessed twice using the same tests: one evaluation was conducted in person, and another one virtually (tele-assessment) by the same trained physical therapist (B.Z.). The interval between evaluations was between 24 to 48 hours. The order of assessments (in person and virtually) and the sequence of administered tests were random. In-person assessments were conducted at the participants’ residences, while tele-assessments were conducted through videoconferencing using the OMPEPE Guideline. Prior to each test, a tutorial video was presented to the individual to explain the steps involved in the test, and any questions or doubts were addressed. All tests included in the online assessment were video recorded. After one month, the first evaluator re-watched the recorded tests to verify intra-rater reliability. For inter-rater reliability, a second physical therapist (C.F.) watched and evaluated the recorded video assessments (as shown in Figure 1). The second physical therapist was blinded to the results. For the online assessments, participants were able to use any device with a camera and internet access (computer, tablet, or cell phone). The materials required to perform the tests, including a cone, measuring tape, and floor marking tape, were supplied by the research team, while participants provided the chair used during the assessments. A digital stopwatch was used to record the duration of each test.

### OMPEPE Guideline

The OMPEPE guideline was developed by our research team to standardize the assessment procedures. It was designed to ensure clarity, reproducibility, and participant safety during each physical test. We developed a guideline to explain each physical test, providing instructions to the participants on:

**O - Objective:** objectives and purposes of the test;
**M - Material:** materials required to carry out the test;
**P - Initial position:** position required to start the test;
**E - Execution:** expected performance for each test, including correct execution, common errors, and compensations;
**P - Final position:** position expected to conclude the test;
**E - Environment:** organization of the testing environment/space to ensure feasibility and safety.

Before the assessments, the researcher asked the participants if they had: (1) taken their medications according to their individual routine; (2) worn appropriate clothing and shoes; (3) eaten and hydrated adequately; (4) refrained from performing any physical exercise prior to the test; (5) ensured all necessary materials were available; and (6) arranged for a companion to be present. The guideline for each test is presented in Appendix A.

### Clinical Measures

The motor section of the Fugl Meyer Assessment (FMA) scale was used to assess the motor impairment of the lower limbs (LL). Severity was stratified according to the score: severe (0–19), moderate (20–28), and mild (scores above 29) (Daly et al., 2011).

### Timed Up and Go Test

The Timed Up and Go (TUG) Test was used to assess the participants’ level of functional mobility (Podsiadlo & Richardson, 1991). The test involves measuring the time it takes for individuals to rise from a chair, walk three meters, navigate around a cone, return to the chair, and sit back down. Participants were instructed to perform the test at their usual pace, choosing the side they would like to walk around the cone. They were also allowed to use a walking device if necessary. The test was conducted three times. The same instructions were given for in-person and tele-assessments.

### Five Times Sit-To-Stand Test

The Five Times Sit-To-Stand Test (5TSTS) was used to evaluate the functional strength and endurance of the lower limbs. Participants were instructed to stand up and sit on a chair with their arms crossed safely and on time, completing the task five times in a row (Whitney et al., 2005). Participants could compensate for the standing transfer by using their upper limbs to assist with the lift if necessary. The test was repeated three times. The same instructions were given for in-person and tele-assessments.

### Functional Reach Test

The Functional Reach Test (FRT) was used to evaluate dynamic balance and functional reach in a standing position. Participants were instructed to stand beside a wall and flex their upper limb at a 90-degree angle. The evaluator marked the initial position of the distal end of the third metacarpal on the wall and then instructed the participant to reach forward as far as possible without touching the wall, using the verbal command “reach as far as you can without taking a step.” The evaluator marked the final position of the distal end of the third metacarpal on the wall. The test was performed three times, and the scores were determined by measuring the difference in centimeters between the initial and final positions (Duncan et al., 1990; Katz-Leurer et al., 2009). If participants were unable to lift the affected arm, they were instructed to perform the reaching movement using their unaffected arm. During tele-assessments, the participant’s caregiver or family member was responsible for marking and measuring the distance between the initial and final positions of the reaching task. The measurement was then shared with the evaluator to determine the participant’s score.

### Safety

Safety was determined by monitoring the incidence of adverse events, including falls or near falls, cardiovascular events, and musculoskeletal injuries. Participants and their family members were instructed on what to do in case there were any issues during the tests.

### Statistical Analysis

Data analysis was performed using SPSS 29.0 software (Statistical Package for the Social Sciences, Inc., Chicago, USA). The Shapiro-Wilk test was used to assess the normality of continuous variables. Data were expressed as mean and 95% confidence intervals (continuous variables) and frequency distribution (categorical variables).

Reliability was measured using the Intraclass Correlation Coefficient (ICC) Test to compare in-person versus synchronous tele-assessment, synchronous versus asynchronous tele-assessment, and asynchronous (rater 1) versus asynchronous (rater 2) tele-assessment. We adopted the following values for reliability: <0.5 poor correlation, between 0.5 and 0.75 moderate correlation, between 0.75 and 0.9 good correlation, and >0.9 excellent correlation. The Wilcoxon test was used to compare in-person versus synchronous tele-assessment. Results were considered significant if p < 0.05. The statistical analysis was conducted by averaging three repetitions for all tests.

## Results

### Participants

**Figure 2:**
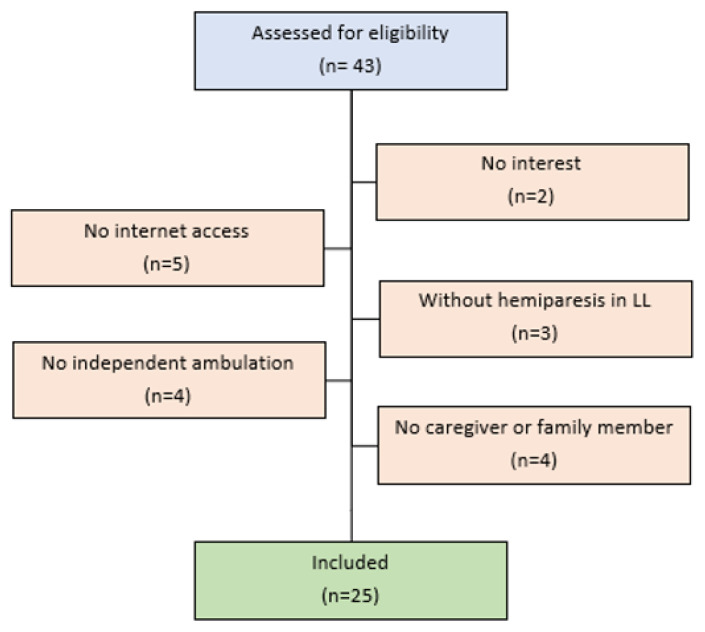
Recruitment Process Flow Chart

Forty-three subjects were recruited for this study. Eighteen subjects were excluded based on eligibility criteria. Twenty-five individuals post-stroke with chronic hemiparesis were included (Figure 2). Participants had moderate to severe motor impairment of the lower limbs. The sample’s characteristics are depicted in Table 1. The detailed results of evaluations are presented in Appendix B.

### In-person Versus Tele-assessment

All participants completed the full evaluation protocol except for one participant who experienced a technical problem (i.e., battery ran out) during the FRT test and was therefore excluded from the analysis. Our analysis revealed that the reliability of all tests, including TUG, 5TSTS, and FRT, ranged from good (ICC > 0.75) to excellent (ICC > 0.90) when comparing in-person and tele-assessments. Moreover, we did not detect any significant difference between the scores obtained from the in-person and tele-assessment methods for these tests (p > 0.05 for all) (Table 2). Furthermore, we did not observe any adverse events during the evaluations.

### Intra-rater and Inter-rater Reliability

Table 3 summarizes the results of two investigations: (a) a comparison between the tele-assessments conducted synchronously and asynchronously by the same rater after one month (to verify intra-rater reliability); (b) a comparison between the tele-assessments conducted asynchronously by the first and second raters (to verify inter-rater reliability). The intra-rater and inter-rater reliability of the TUG and 5TSTS tests were excellent (ICC > 0.90). However, we were unable to evaluate the intra-rater and inter-rater reliability of the FRT due to the measurement method used in tele-assessments. During the tele-assessment, the caregiver or family member was instructed to conduct and report the test results to the evaluator, making it impossible to assess the measurement consistency during the review after the evaluation.

## Discussion

This study investigated the reliability and safety of a tele-assessment protocol for evaluating mobility and balance in individuals with chronic hemiparesis following stroke. We developed a guideline called OMPEPE to standardize the online evaluation process. Our results demonstrated a high level of agreement between online and in-person assessments and excellent intra-rater and inter-rater reliability for the synchronous and asynchronous online format. Notably, no adverse events occurred during the experiment, such as falls or near falls. Therefore, our findings suggest that tele-assessment is safe and reliable for evaluating balance and mobility when using a systematized guideline protocol for people living with chronic post-stroke impairments.

We found a strong correlation between in-person and tele-assessment for TUG, 5TSTS, and FRT tests when following the OMPEPE guideline. Similarly, the online assessments demonstrated excellent intra-rater and inter-rater reliability when different examiners scored the same phenomenon (ICC > 0.90). However, examiners evaluated and scored tests’ performance in different formats (synchronous and asynchronous), and this could be an important bias. That is why following the OMPEPE guideline might be valuable when different examiners perform the same test by tele-assessment. When comparing our results to the healthy population, a previous study found good to moderate inter-rater reliability for TUG and 5TSTS (Bird et al., 2022). A systematic review that assessed the effectiveness of physiotherapy assessments delivered through telehealth found that assessments requiring observation and/or timing, such as the TUG test, had good feasibility compared to in-person assessments (Zischke et al., 2021).

Although tele-assessment is a convenient way to monitor patients remotely, some barriers and limitations must be considered. In our study, we experienced delays in some tele-assessments due to technical difficulties faced by some participants and their companions, particularly those who were older or had lower levels of education. Another significant barrier was related to poor internet connectivity, such as disconnection or delays. In such cases, the evaluation was restarted. For future studies, we suggest that the quality of the internet be tested beforehand, as well as the technical knowledge of the participants or their caregiver/family member regarding their familiarity with managing devices and online platforms. Moreover, the home environment presented some challenges, including noise, visual distractions, and other external noises. The limited physical space inside the home sometimes requires evaluations to be carried out in unconventional places such as hallways or garages. However, despite these challenges, both in-person and tele-assessments were conducted similarly. Nonetheless, participants’ perceptions of tele-assessments were not investigated in this study, and thus, the barriers and facilities we encountered should be interpreted with caution.

Future studies should include larger sample sizes and employ more robust statistical methods to enhance the generalizability of findings. Another promising avenue is the expansion of the OMPEPE guideline to other neurological populations and health conditions. Research should further investigate its applicability and validity across diverse clinical contexts, with the ultimate goal of developing standardized tele-assessment protocols that ensure accuracy, safety, and accessibility for people with limited mobility or those living in remote areas.

Our study highlights the reliability of tele-assessment for evaluating mobility and balance in people with chronic post-stroke impairments. The findings provide evidence that implementing and following a systematic guideline for tele-assessment may promote accurate and safe evaluations. Tele-assessment following the OMPEPE guideline demonstrated excellent intra-rater and inter-rater reliability, indicating that tele-assessment can be a reliable alternative to in-person assessments when properly conducted. Our results may have important implications for the care of people post-stroke and other neurological populations. Tele-assessment may represent a valuable tool for monitoring the mobility and balance in people who face barriers to accessing in-person healthcare services. This study’s findings also encourage the development of tele-assessment guidelines and protocols for other health conditions, potentially improving access to care for patients with limited mobility or those living in remote areas.

## Figures and Tables

**Table 1:** Sample’s Characteristics

Variables		Post-stroke individuals (n=25)

Age (years)		60.9 (55.70–66.13)

Gender (Male/Female)		18/7

Educational level	Basic education	5
	High school	4
	Higher Education	16

Time since stroke (months)		55 (35.51–74.48)

Stroke type (Ischemic/Hemorrhagic)		17/8

Affected side (Right/Left)		8/17

Mini-Mental State Examination		27.3 (26.12–28.43)

FMA-LL (Severe/Moderate)		13/12

*Note*. Data are expressed as frequencies, means, and standard deviations. FMA-LL: Fugl-Meyer Assessment - lower limb; n: number of participants.

**Table 2:** In-person versus Tele-assessment

Test	In-person	Tele-assessment (synchronous)	P-value	ICC (95% CI)
TUG (s)	40.36 (26.46–54.26)	40.10 (26.25–53.95)	0.861	0.98
5TSTS (s)	24.47 (20.52–28.42)	25.22 (20.91–29.54)	0.527	0.95
FRT (cm)	19.56 (16.72–22.40)	18.96 (15.43–22.49)	0.742	0.84

*Note*. Data are expressed as means and standard deviations. TUG: Timed Up and Go Test; 5TSTS: 5-Time Sit-To-Stand Test; FRT: Functional Reach Test. ICC: Intraclass Correlation Coefficient.

**Table 3:** Intraclass Correlation Coefficient (ICC) for Inter-rater and Intra-rater Reliability

Assessment	Rater 1 online synchronous	Rater 1 online asynchronous	ICC (95% CI)	Rater 2 online asynchronous	ICC (95% CI)
TUG (s)	40.10 (26.25–53.95)	40.00 (26.24–53.77)	1.00 (1.00–1.00) a	39.82 (26.18–53.45)	1.00 (1.00–1.00) b
5TSTS (s)	25.22 (20.91–29.54)	25.25 (20.94–29.56)	1.00 (1.00–1.00) a	25.15 (20.87–29.44)	1.00 (1.00–1.00) b

*Note*. Data are expressed as means and standard deviations. TUG: Timed Up and Go Test; 5TSTS: 5-Time Sit-To-Stand Test; ICC: Intraclass Correlation Coefficient. CI: confidence interval.

a – intra-rater reliability.

b – inter-rater reliability.

## Data Availability

The authors confirm that the data supporting the findings of this study are available within the article [and/or] its supplementary materials.

## Appendix A OMPEPE Guideline

**Table:**

*Timed Up and Go Test*	
**O**	We will administer this test, which is called Timed Up and Go, to evaluate your functional mobility, which refers to your ability to move around to perform everyday activities.
**M**	You will require the following materials to conduct the test: a measuring tape, a chair, and a cone. The cone should be placed 3 meters away from the chair.
**P**	To begin the test, you must be in a seated position on the chair.
**E**	When I give you the command, stand up from the chair, walk the distance of 3 meters, go around the cone, come back to the starting point, and then sit down on the chair at your usual or comfortable speed.
**P**	At the end of the test, you should be sitting in the chair again.
**E**	The testing area must be clear of any obstacles or objects that could disrupt the test or draw your attention away. The test can be conducted indoors, in a hallway, or in a garage, as long as the area is at least 3 meters in length.
Observation: We will perform the test three times to ensure accuracy. You can use devices to aid during the test, such as crutches if necessary.	

**Table:**

*Five Times Sit to Stand Test*	
**O**	We will administer this test, which is called the Five Times Sit to Stand Test, to assess the strength and endurance of your legs.
**M**	The only item required for this test is a chair.
**P**	To initiate the test, make sure you are seated on the chair, with your feet firmly on the floor and your arms crossed in front of your chest.
**E**	When I give you the command, you should stand up and sit down five times as fast as you can.
**P**	At the end of the test, you should be sitting in the chair again.
**E**	The testing area must be clear of any obstacles or objects that could disrupt the test or draw your attention away. The chair must be placed on the floor stably and safely.
Observation: We will perform the test three times to ensure accuracy.	

**Table:**

*Functional Reach Test*	
**O**	We will administer this test, which is called the Functional Reach Test, to assess your dynamic balance, which is your ability to maintain balance while moving.
**M**	You will require the following materials to conduct the test: a measuring tape and sticky tape.
**P**	To begin the test, stand next to a wall, with your side facing the wall, and make sure that you do not touch the wall during the test.
**E**	Please flex your arm to a 90-degree angle when I give you my first command. Your assistant will mark the farthest point reached by your third finger on the wall. Once I give you the second command, you should lean your body forward, reaching as far as you can without taking a step or touching the wall. Your assistant will mark the new location where your third finger reaches. Your assistant should then measure the distance in centimeters between the starting and final positions.
**P**	Once you have reached as far as possible, please return to the starting position in a relaxed manner, with your side facing the wall.
**E**	The testing area must be clear of any obstacles or objects that could disrupt the test or draw your attention away.
Observation: Please perform the reaching movement with your affected arm. If you are unable to do so, you may use your unaffected arm instead. If you are unable to lift either arm, simply lean your torso forward, and we will measure the distance covered by your shoulder. We will perform the test three times to ensure accuracy.	

## Appendix B Detailed Results Obtained from Evaluations Conducted In-person and Through Tele-assessment

**Table:**

			IN-PERSON ASSESSMENT			ON-LINE SYNCHRONOUS ASSESSMENT			PHYSICAL THERAPIST 2 REVIEW		
	Participants	FMA-MI	TUG (s)	5TSTS (s)	FRT (cm)	TUG (s)	5TSTS (s)	FRT (cm)	TUG (s)	5TSTS (s)	FRT (cm)
MODERATE	1	27	24,21	31,27	18,50	26,19	27,42	15,33	26,13	27,87	15,33
	2	27	11,38	10,42	37,67	12,13	13,91	37,83	12,56	13,87	37,83
	3	27	17,96	30,21	11,50	18,60	25,89	11,00	19,06	25,66	11,00
	4	23	14,03	16,11	12,33	13,96	14,09	14,00	14,54	14,15	14,00
	5	23	15,38	19,01	21,83	17,89	27,21	11,00	18,07	27,06	11,00
	6	23	31,36	23,53	20,33	30,26	24,23	19,00	30,21	24,46	19,00
	7	22	18,46	24,47	25,33	18,89	22,96	31,83	18,89	22,84	31,83
	8	22	10,31	9,62	12,17	10,03	11,89	18,00	11,01	12,85	18,00
	9	22	11,56	11,79	25,67	12,25	14,06	30,00	11,56	14,51	30,00
	10	20	22,67	27,85	22,00	19,24	27,37	12,00	18,77	29,93	12,00
	11	20	20,34	15,26	27,00	19,48	16,67	29,33	19,07	15,91	29,33
	12	20	21,09	20,17	18,67	19,86	21,18	13,67	20,14	21,32	13,67
SEVERE	1	19	48,26	32,70	12,00	52,63	34,54	20,17	52,06	34,38	20,17
	2	19	13,51	15,18	28,83	15,15	16,48	17,67	15,41	16,54	17,67
	3	17	57,33	25,12	19,75	53,81	17,67	20,00	54,03	17,18	20,00
	4	16	98,35	25,24	16,00	100,81	30,80	25,00	99,02	30,33	25,00
	5	15	19,59	35,04	23,33	21,89	47,73	26,00	21,87	47,07	26,00
	6	15	87,85	22,79	23,33	104,91	23,88	27,17	105,47	23,46	27,17
	7	15	111,49	24,29	-	82,25	22,89	-	82,03	22,68	-
	8	14	98,20	45,72	14,67	81,40	49,63	9,67	77,96	49,60	9,67
	9	14	35,76	34,67	16,33	36,59	28,30	16,67	35,72	27,00	16,67
	10	14	60,87	21,37	17,17	54,66	22,58	9,25	53,57	22,14	9,25
	11	13	97,23	25,98	10,67	83,33	22,70	10,00	83,15	22,03	10,00
	12	13	27,18	22,12	23,83	24,84	20,68	24,33	25,17	20,10	24,33
	13	13	105,95	41,75	10,67	113,74	43,64	6,25	112,26	43,50	6,25

*Note*. TUG: Timed Up and Go Test; 5TSTS: Five Times Sit-To-Stand Test; FRT: Functional Reach Test
